# Neuromonitoring, neuroimaging, and neurodevelopmental follow-up practices in neonatal congenital heart disease: a European survey

**DOI:** 10.1038/s41390-022-02063-2

**Published:** 2022-04-12

**Authors:** Maria Feldmann, Cornelia Hagmann, Linda de Vries, Vera Disselhoff, Kuberan Pushparajah, Thushiha Logeswaran, Nicolaas J. G. Jansen, Johannes M. P. J. Breur, Walter Knirsch, Manon Benders, Serena Counsell, Bettina Reich, Beatrice Latal

**Affiliations:** 1 Child Development Centre, University Children’s Hospital Zurich, Zurich, Switzerland; 2 Children’s Research Centre, University Children’s Hospital Zurich, Zurich, Switzerland; 3 Department of Neonatology and Pediatric Intensive Care, University Children’s Hospital Zurich, Zurich, Switzerland; 4 Utrecht Brain Center, UMC Utrecht, Utrecht University, Utrecht, The Netherlands; 5 Pediatric Cardiology Department, Evelina Children’s Hospital London, London, UK; 6 Centre for the Developing Brain, School of Biomedical Engineering and Imaging Sciences, King’s College London, London, UK; 7 Pediatric Heart Center, University Hospital Giessen, Justus-Liebig-University Giessen, Giessen, Germany; 8 Department of Pediatric Intensive Care, Wilhelmina Children’s Hospital, UMC Utrecht, Utrecht, The Netherlands; 9 Department of Pediatrics, Beatrix Children’s Hospital, University Medical Center Groningen, Groningen, The Netherlands; 10 Department of Pediatric Cardiology, Wilhelmina Children’s Hospital, UMC Utrecht, Utrecht, The Netherlands; 11 Pediatric Cardiology, Pediatric Heart Center, University Children’s Hospital Zurich, Zurich, Switzerland; 12 Department of Neonatology, Wilhelmina Children’s Hospital, UMC Utrecht, Utrecht, The Netherlands; 13 Pediatric Cardiology and Congenital Heart Disease, German Heart Centre Munich, Munich, Germany

## Abstract

**Background:**

Brain injury and neurodevelopmental impairment remain a concern in children with complex congenital heart disease (CHD). A practice guideline on neuromonitoring, neuroimaging, and neurodevelopmental follow-up in CHD patients undergoing cardiopulmonary bypass surgery is lacking. The aim of this survey was to systematically evaluate the current practice in centers across Europe.

**Methods:**

An online-based structured survey was sent to pediatric cardiac surgical centers across Europe between April 2019 and June 2020. Results were summarized by descriptive statistics.

**Results:**

Valid responses were received by 25 European centers, of which 23 completed the questionnaire to the last page. Near-infrared spectroscopy was the most commonly used neuromonitoring modality used in 64, 80, and 72% preoperatively, intraoperatively, and postoperatively, respectively. Neuroimaging was most commonly performed by means of cranial ultrasound in 96 and 84% preoperatively and postoperatively, respectively. Magnetic resonance imaging was obtained in 72 and 44% preoperatively and postoperatively, respectively, but was predominantly reserved for clinically symptomatic patients (preoperatively 67%, postoperatively 64%). Neurodevelopmental follow-up was implemented in 40% of centers and planned in 24%.

**Conclusions:**

Heterogeneity in perioperative neuromonitoring and neuroimaging practice in CHD in centers across Europe is large. The need for neurodevelopmental follow-up has been recognized. A clear practice guideline is urgently needed.

**Impact:**

- There is large heterogeneity in neuromonitoring, neuroimaging, and neurodevelopmental follow-up practices among European centers caring for neonates with complex congenital heart disease.
- This study provides a systematic evaluation of the current neuromonitoring, neuroimaging, and neurodevelopmental follow-up practice in Europe.
- The results of this survey may serve as the basis for developing a clear practice guideline that could help to early detect and prevent neurological and neurodevelopmental sequelae in neonates with complex congenital heart disease.

## Introduction

In Europe, the prevalence of congenital heart disease (CHD) is 8 per 1000 live births.^1^ Over the past decades, survival rates of children with complex CHD have significantly improved due to advances in neonatal and intensive care medicine and surgical techniques.^2^ However, this patient population continues to be at significant risk for neurodevelopmental sequelae throughout their life course^3,4^ This may exert long-lasting adverse effects on the individuals, their families, and society.^5^

As potential underlying mechanisms, the risk for abnormal intrauterine brain development and neonatal brain injury has long been recognized in this population, with small focal strokes and punctate white matter lesions being the most frequently observed types of brain lesions.^6–14^ Numerous studies have shown that neuroimaging and neuromonitoring techniques can be valuable tools in the early detection of neonatal brain injury, identification of risk for later neurodevelopmental sequelae, and help optimize perioperative care to attenuate and modify risk factors.^11,15–18^ Despite this evidence, there is no clear practice guideline or standardized protocol on how to monitor neonates with complex CHD in the vulnerable perioperative period.

In contrast, there is consensus on the need for neurodevelopmental follow-up for children with complex CHD and recently recommendations for comprehensive neurodevelopmental follow-up programs for preschool^19^ and school-age children^20^ have been issued. However, little is known about the implementation of these guidelines in neonatal cardiac surgery centers across Europe.

The European Association Brain and Congenital Heart Disease (ABC) Consortium is a multicenter, multidisciplinary group, financially supported by the European Society for Paediatric Research, that aims to promote research in the field of brain development in CHD children and, thereby, improve the neurodevelopmental outcome of infants with severe CHD.^21^ With this survey, we aimed to obtain detailed information on neuromonitoring practices in European centers caring for neonates with CHD. We designed and distributed an online survey inquiring about the use and timing of neuromonitoring and neuroimaging tools in pediatric cardiac surgical centers. Furthermore, we obtained information on the implementation of neurodevelopmental follow-up programs and the general interest in a European neurodevelopmental outcome registry. The results of this survey may serve as the basis for the development of an expert panel recommendation on neuromonitoring, neuroimaging, and neurodevelopmental follow-up in CHD patients undergoing neonatal cardiac surgery.

## Methods

We conducted a structured online survey on the current implementation of neuromonitoring, neuroimaging, and neurodevelopmental follow-up in infants with congenital heart disease who require cardiopulmonary bypass surgery. The questionnaire included details on preoperative, intraoperative, and postoperative neuromonitoring modalities, preoperative and postoperative neuroimaging, measurement of postoperative biochemical markers, and the implementation of neurodevelopmental follow-up and data entry into a register. Furthermore, we investigated the interest in participating in a European neurodevelopmental outcome registry.

The survey was constructed and designed with the professional and freely accessible online survey tool available on https://www.soscisurvey.de/ (last accessed March 2021). As there was no standardized questionnaire available on perioperative neuromonitoring and neuroimaging, the content and design of the questionnaire were jointly agreed upon by consensus from the European ABC Consortium. Furthermore, we piloted and modified the questionnaire within our study group. The questionnaire was directly distributed to pediatric cardiac surgical centers, the European Brain Club, the Association for European Pediatric and Congenital Cardiology (AEPC), and personal contacts by members of the consortium. Furthermore, the questionnaire was sent to all members of the European Society for Paediatric Research via the monthly newsletter. The survey can be found as Supplementary Fig. 1. Respondents were asked to voluntarily identify their institution and provide contact information for potential follow-up questions. If multiple reports of the same institution were identifiable, centers were contacted to reach a consensus on their entries, or the most complete entry was kept. Data were collected between April 2019 and June 2020. Only valid questionnaire responses were considered for further analysis. Valid entries were defined as respondents reaching at least page 6 of 19, i.e., at least reaching questions on preoperative neuromonitoring. Furthermore, we defined a rate of <60% missing entries as a valid response cut-off to exclude cases in which respondents just viewed the questionnaire without filling it in. This research project was deemed exempt from the requirement for approval by the ethics committee.

### Statistical analysis

The evaluation of the survey results was of descriptive nature. Categorical variables were reported as frequencies and percentages. Free text comments were qualitatively summarized.

## Results

### General characteristics of the responding centers

**Fig. 1:**
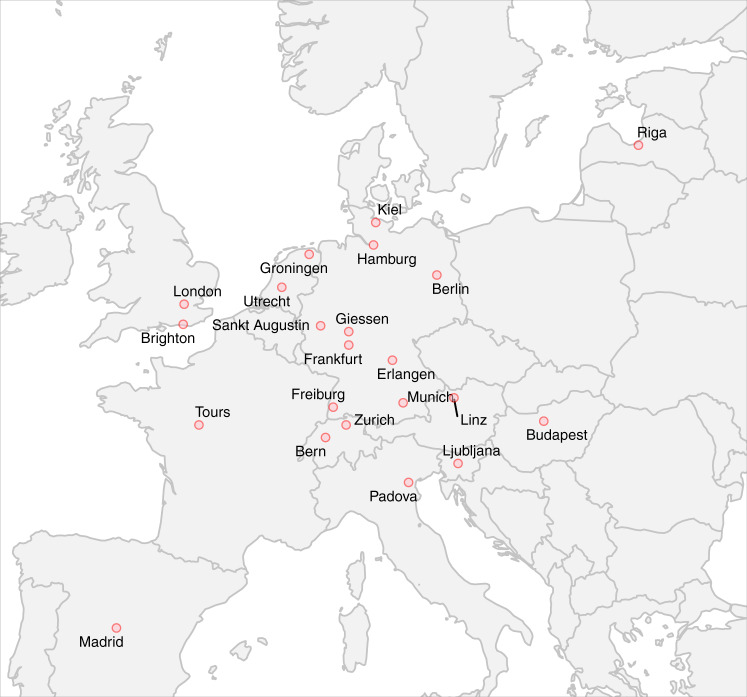
Map plot illustrating the distribution of the 25 European sites that provided a valid questionnaire response.

During the survey period, 25 European centers provided a valid response to the questionnaire (*n* = 22 excluded for aborting the questionnaire before page 6; *n* = 3 excluded for >60% missing entries). Among these, 23/25 centers completed the questionnaire to the last page. The completion rate, i.e., the number of complete questionnaires divided by started questionnaires measured by clicks on the link was 46% (23/50). For the geographical distribution of the 25 centers across Europe who provided a valid entry, see Fig. 1. At the centers, the questionnaire was filled in by pediatric cardiologists in 40% (10/25), neonatologists in 20% (5/25), others (e.g., pediatric neurologists, pediatric radiologists, developmental pediatricians, pediatric and congenital cardiac surgeons) in 12% (3/25), and pediatric intensivists in 8% (2/25) of cases. Multiple subspecialties were indicated by 20% (5/25).

**Fig. 2:**
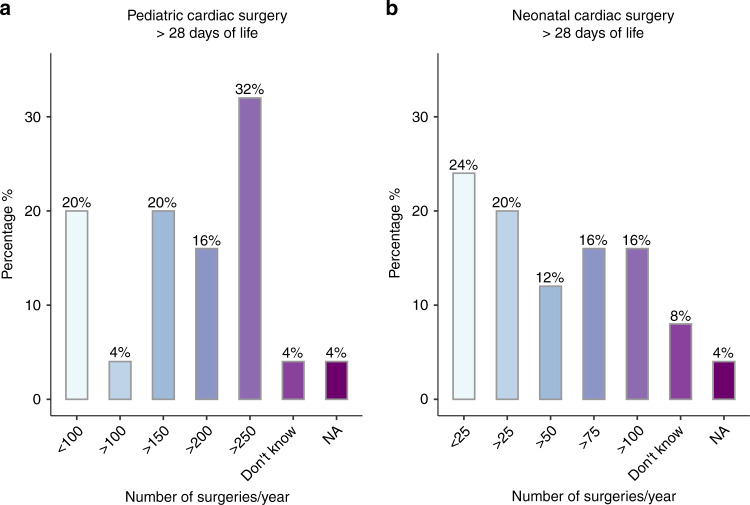
Number of pediatric/neonatal cardiac surgeries. Bar plot illustrating the distribution of yearly number of (**a**) pediatric and (**b**) neonatal cardiac surgeries at the responding centers.

The majority of centers (56% (14/25)) indicated that preoperative care in infants with CHD was carried out in different units depending on the age and clinical status of the patients. In 20% (5/25), treatment was carried out in a neonatology unit, in 16% (4/25) in a cardiology unit, and in 8% (2/25) in a pediatric intensive care unit. Regardless of the type of unit, 88% (22/25) of centers identified their unit as an intermediate or intensive care ward. In 32% (8/25) of responding centers >250 pediatric cardiac surgeries (age 0–16 years) were performed each year (Fig. 2a). In contrast, only 16% (4/25) of centers performed >100 neonatal cardiac surgeries with cardiopulmonary bypass surgery (<28 days of life) each year, whereas 24% (6/25) of centers performed <25 neonatal surgeries each year (Fig. 2b). The majority of centers (72% (18/25)) indicated performing the hybrid procedure (i.e., stenting of the arterial duct and banding of the pulmonary arteries^22^) in neonates with hypoplastic left heart syndrome in <25 neonates per year. In 16% (4/25), >25 hybrid procedures per year were performed, whereas 12% (3/25) did not know or answer the question.

### Preoperative neuromonitoring practice

**Fig. 3:**
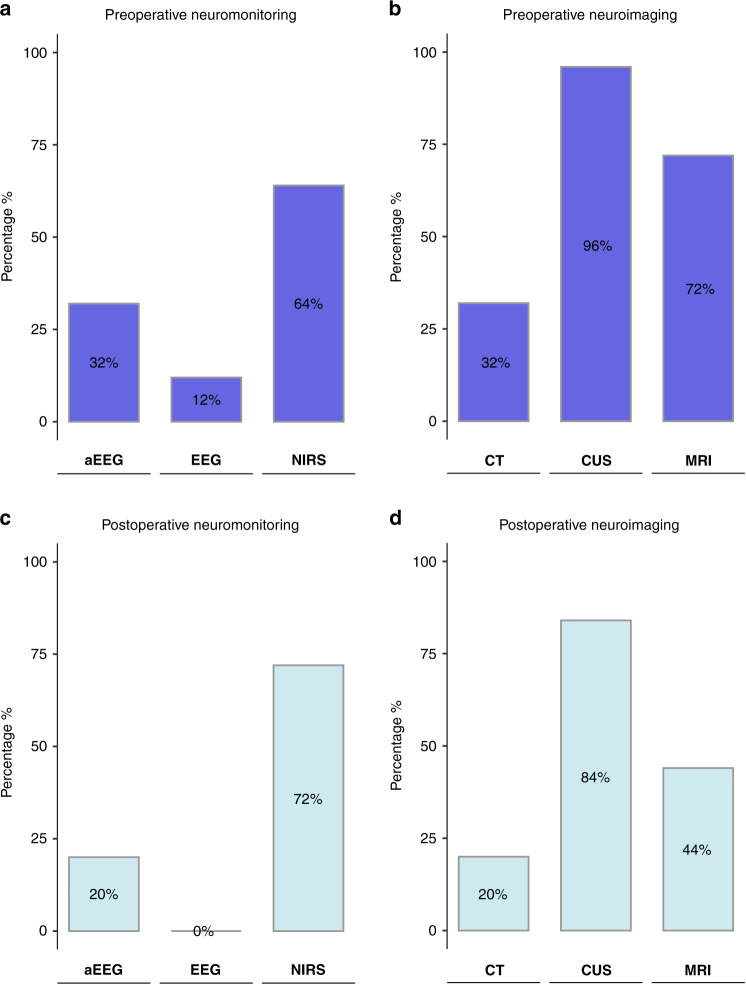
Use of pre- and postoperative neuromonitoring and neuroimaging tools. Bar plot showing the percentages of preoperatively and postoperatively used neuromonitoring and neuroimaging tools. aEEG amplitude-integrated electroencephalography, NIRS near-infrared spectroscopy, EEG electroencephalography, CT computer tomography, CUS cranial ultrasound, MRI magnetic resonance imaging.

Regarding their preoperative neuromonitoring practice for neonates prior to cardiopulmonary bypass surgery, the majority used near-infrared spectroscopy (NIRS) 64% (16/25), while only 32% (8/25) used amplitude-integrated electroencephalography (aEEG) and 12% (3/25) used continuous video electroencephalography (cEEG) (Fig. 3a). Furthermore, 28% (7/25) of the responding centers indicated that no neuromonitoring tool was used prior to neonatal cardiac surgery. When using NIRS, a neonatal sensor as opposed to a pediatric/adult sensor was most frequently used (93.8% (15/16)). Among centers using aEEG, 2-channel aEEG as opposed to 1-channel aEEG was used in 75% (6/8). In those centers which indicated that neonatal preoperative neuromonitoring was performed, it was most often performed on a routine clinical basis (72.2% (13/18)), or on clinical indication (22.2% (4/18)), and only in 5.6% (1/18) merely for research purposes. Most centers performed a preoperative neurological examination only in cases of clinical concern 56% (14/25). Only a few centers performed the examination on a clinical routine basis (8% (2/25)) or as part of a research protocol (16% (4/25)). The examination was most often performed by a child neurologist or neonatologist (36% (9/25) and 40% (10/25)).

### Preoperative neuroimaging practice

**Fig. 4:**
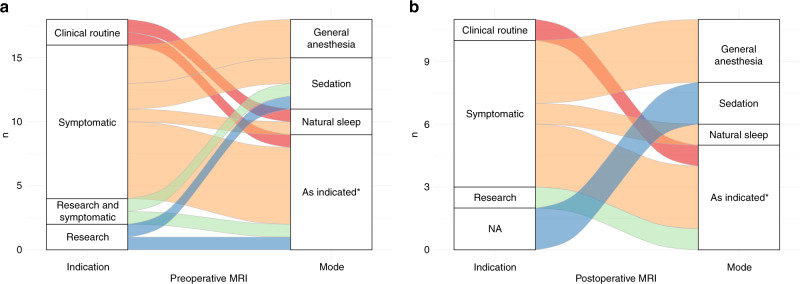
Use of pre- and postoperative MRI. Alluvial diagram illustrating the indication and mode of sedation/performance of preoperatively and postoperatively obtained cerebral magnetic resonance images (MRI). *As indicated: modality depends on the clinical condition.

Preoperative neuroimaging was used in almost all centers (96% (24/25)). Cranial ultrasound was used by 96% (24/25), magnetic resonance imaging (MRI) by 72% (18/25), and computer tomography (CT) by 32% (8/25) of responding centers (Fig. 3b). In the 18 centers performing MRI, it was mostly done for clinically symptomatic patients (67% (12/18)). In 33% (6/18) of centers, preoperative MRI was performed routinely either as a clinical routine (11% (2/18)) or for research purposes (22% (4/18)). One of the centers performing clinical routine MRI indicated that this was done in all patients undergoing cardiopulmonary bypass surgery, whereas the other center specifically targeted patients with transposition of the great arteries, aortic arch interruption, or hypoplastic left heart syndrome. Only 17% (3/18) of centers reported that routine clinical or research MRI was also performed in the intensive care unit. In free-text comments, respondents specified that MRI in intensive care unit patients was reserved only for symptomatic patients due to limited resources. MRI was either performed using sedation (22% (4/18)), in natural sleep with a feed and wrap technique (11% (2/18)), using general anesthesia (17% (3/18)) or a combination of modalities depending on the clinical situation (50% (9/18)) (Fig. 4a). We did not further inquire about the circumstances under which cranial ultrasound or CT was performed.

### Intraoperative neuromonitoring practice

Intraoperative neuromonitoring was performed using NIRS in 80% (20/25), aEEG in 16% (4/25), bispectral index in 8% (2/25), and cEEG in 4% (1/25). No intraoperative neuromonitoring was performed in 8% (2/25), and no response was given by 12% (3/25) of questionnaire participants. The majority of centers did not administer neuroprotective agents (e.g., steroids, allopurinol, nitric oxide, mannitol, corticosteroids) (64% (16/25)) intraoperatively, 8% (2/25) did not know and 8% (2/25) did not answer the question.

### Postoperative measurement of biochemical markers of neuronal injury

Postoperatively, most centers did not measure biomarkers of neuronal injury (64% (16/25)) or did not know (8% (2/25)) or answer the question (8% (2/25)). Only a few centers indicated measuring parameters such as neuron-specific enolase, protein S100, or glial fibrillary acidic protein (20% (5/25)) to assess neuronal injury post-surgery.

### Postoperative neuromonitoring practice

At least one method of postoperative neuromonitoring was utilized in 72% (18/25) of centers, most commonly NIRS (72% (18/25)) and aEEG (20% (5/25) (Fig. 3c). Both tools in combination were used in 20% (5/25) of centers. A proportion of 20% (5/25) said they did not perform postoperative neuromonitoring or did not provide a response (8% (2/25)). Half of the respondents (48% (12/25)) indicated that a postoperative neurological examination was only performed in patients with clinical concerns. Furthermore, 24% (6/25) of centers indicated that no neurological examination was being routinely performed. A proportion of 20% (5/25) of responding centers indicated that they did perform a neurological examination, which was done in 80% (4/5) as part of a research protocol and only in one center (20% (1/5)) as part of clinical routine. 8% (2/25) did not respond to the question.

### Postoperative neuroimaging practice

Postoperatively, the majority of responding centers (88% (22/25)) used at least one neuroimaging modality, only 4% (1/25) did not use any neuroimaging or did not respond to the question (8% (2/25)). Cranial ultrasound was used by 84% (21/25), MRI by 44% (11/25), and CT by 20% (5/25) of centers (Fig. 3c). Of those centers performing MRI postoperatively, it was most commonly acquired in symptomatic patients only (64% (7/11)), followed by a combination of symptomatic patients and based on a research protocol (18% (2/11)). Only one center performed MRI only for research purposes or as part of clinical routine respectively (9% (1/11)). Specifically, susceptibility weighted images to detect potential hemorrhagic lesions were acquired in 27% (3/11) of the centers, whereas 18% (2/11) additionally acquired venograms to detect sinovenous thromboses. A proportion of 27% (3/11) of centers performed postoperative MRI using general anesthesia, 18% (2/11) used sedation or a combination of natural sleep, sedation, and general anesthesia in 55% (6/11) (Fig. 4b). We did not further inquire about the circumstances under which cranial ultrasound or CT was performed.

### Neurodevelopmental follow-up

Almost half of the responding centers (40% (10/25)) indicated having a follow-up program in place for children with CHD after cardiopulmonary bypass surgery, and 24% (6/25) were planning to implement one. In contrast, 28% (7/25) of centers did not have a follow-up program and 8% (2/25) did not respond to the question. Follow-up data were collected in a register in 32% (8/25); 32% (8/25) did not collect follow-up information and the remaining centers (36% (9/25)) did not respond to the question. The majority of centers (88% (22/25)) said they were interested in joining a European neurodevelopmental outcome register for neonates with CHD.

## Discussion

In this European survey on perioperative neuromonitoring, neuroimaging, and neurodevelopmental follow-up in neonates undergoing cardiopulmonary bypass surgery for complex CHD, we found evidence for very heterogeneous practices in cardiac surgical centers across Europe.

Preoperatively, intraoperatively, and postoperatively NIRS was implemented in 2/3 of responding centers and was thus the most commonly used tool for neuromonitoring at all three inquired time points. Cardiac surgery is known to be associated intraoperatively and postoperatively with altered cerebral hemodynamic evident on NIRS monitoring.^17^ While NIRS is a valuable tool to monitor these perioperative changes and guide perioperative and intraoperative intensive care management, there are conflicting results on the predictive values of those parameters for brain injury and later neurodevelopment as reviewed by Zaleski and colleagues^17^ and reported in recent publications.^23^

In contrast, aEEG was only used in 20–32% of centers in the perioperative period. However, cerebral function monitoring by means of aEEG is recommended in neonates undergoing early surgery for CHD for surveillance and treatment of seizures.^24^ Furthermore, studies have shown that beyond seizure detection aEEG can inform the intensive care team about the risk for neonatal brain injury and long-term outcome prognosis. Preoperatively, an abnormal background pattern on aEEG was associated with neonatal brain injury,^25^ whereas in another study postoperative abnormal brain activity on aEEG (i.e., abnormal background pattern or ictal discharges) was associated with a fourfold increase in the risk for postoperative brain injury.^15^ While the predictive value of aEEG for the neurodevelopmental outcome has been well established in the population of infants with hypoxic–ischemic encephalopathy, first evidence showed a promising predictive value also in the postoperative cardiac population. Postoperatively, delayed recovery of background pattern on aEEG and lack of return to normal sleep–wake cycling has been shown to be associated with poor neurodevelopmental and cognitive outcomes at 2 and 4 years of age.^16,26^ A more systematic use and widespread implementation of this technique in perioperative infants with CHD could help to further improve the diagnostic and prognostic leverage of this tool.

Almost all centers used preoperative neuroimaging, with cranial ultrasound and MRI as the most commonly used modalities. However, MRI was predominantly only used in clinically symptomatic patients and rarely in patients cared for in the intensive care unit. The same was found for postoperative neuroimaging practices. Nevertheless, there is a large body of evidence demonstrating that perioperative brain injury is common in CHD^21^ and is clinically silent in the majority of cases.^14^ Additionally, studies have linked MRI-detected perioperative brain lesions with later adverse neurodevelopmental outcome^18,27^ underlining the potential predictive value of the tool. Furthermore, patients that require prolonged preoperative or postoperative care in an intensive care unit might be at the highest risk for neonatal brain injury, which can be missed, if a timely MRI is not available to these patients.^21,28^ Thus, the routine implementation of MRI-based neuroimaging preoperatively or postoperatively seems of additional value in the cardiac population. For cranial ultrasound, only one preoperative study was performed showing no predictive value for the later outcome. However median age at examination was 3 days of life and further studies are lacking.^29^

There were few centers performing MRI with sedation or general anesthesia as opposed to natural sleep. However, sedation and general anesthesia are associated with additional risks for patients and require further complex logistics and personnel resources. Thus institutional neuroimaging protocols that require anesthesia might pose a significant barrier to using MRI more frequently and on a clinical routine basis. However, protocols for performing neonatal MRI in natural sleep have been tested and validated.^30,31^ Furthermore, a number of studies at different sites have shown that MRI in natural sleep is feasible and can yield images of high quality in neonates with CHD.^14,32,33^ Exchanging experience in performing MRI in natural sleep among European pediatric heart centers could increase the use of perioperative MRI and help leverage this tool for the detection of brain injury in the vulnerable population of neonates with CHD.

In neonatal at-risk populations, a standardized neonatal neurological examination or assessment of General Movements has been shown to be a key tool in the early identification and prediction of motor impairments such as cerebral palsy.^34^ For the CHD population, studies have shown that standardized assessments such as the Neonatal Intensive Care Unit Network Neurobehavioral Scale,^35,36^ the Einstein Neonatal Neurobehavioral Assessment Scale^37,38^ or a General Movements assessment^39^ can reveal neurological and neurobehavioral abnormalities that are already evident in the preoperative and postoperative neonatal period and are associated with brain volumetric measurements on MRI.^38^ Furthermore, the General Movements assessment has been shown to have a good predictive value for later cognitive and neuromotor outcome.^40,41^ Thus, performing a standardized neurological assessment before hospital discharge is a crucial prerequisite to counsel parents, implement early support, and tailor follow-up and should be standard of care in all centers caring for neonates with complex CHD. However, among the responding centers across Europe, a postoperative neurological examination has been reported to be performed in only 20% of infants after cardiopulmonary bypass surgery, possibly leading to many missed opportunities of implementing early support for the child and their family. This current practice is in contrast to the recently published recommendations from the Cardiac Neurodevelopmental Outcome Collaborative on neurodevelopmental evaluation strategies for young children with CHD, which suggests performing a neurobehavioral consultation prior to hospital discharge in all infants with CHD.^19^ In line with other current recommendations^19,20^ the importance of neurodevelopmental follow-up in this vulnerable population has been recognized across Europe, with 2/3 of responding centers having already implemented or are planning to implement a structured follow-up program. However, outcome data collection is often not performed uniformly or systematically, which is a requirement for quality control and comparison across centers. Accordingly, the majority of participating centers have formulated the wish to join a European CHD outcome register. Furthermore, an adaptation of follow-up guidelines to European or even country-specific health care characteristics might be necessary to ensure broad and comprehensive implementation of structured follow-up programs, as a recent Canadian analysis demonstrated.^42^ To further investigate this, an in-depth evaluation of center-specific follow-up programs in European pediatric heart centers is warranted in future studies.

### Limitation

This study has several limitations. As there was no standardized questionnaire available, we used a set of questions that were selected based on expert opinions from the European ABC Consortium. The questionnaire was not formally validated within an independent test set. Although we tried to reach out to all European heart centers by distributing our questionnaire via multiple channels, we were only able to cover a fraction of all centers caring for neonates with complex CHD. We found a dense and satisfiable response rate in central Europe, whereas many other (e.g., Scandinavian or eastern European) countries were missed. Considering that there are 32 national delegates in the AEPC, our sample is likely only representative of a small fraction of European centers and potentially overestimates the use of perioperative neuromonitoring tools and implementation of follow-up in Europe. The completion rate of the questionnaire relative to started questionnaires by clicks on the survey link was relatively low. However, sending out impersonalized links did not allow us to filter out multiple clicks on the link by the same respondents.

## Conclusion

There is large heterogeneity in perioperative neuromonitoring and neuroimaging practice in centers across Europe. Despite evidence for the value of aEEG, MRI, and clinical examination in detecting and predicting adverse neurological outcomes in CHD a consistent and routine implementation in pediatric heart centers across Europe is lacking. The need for neurodevelopmental follow-up has been widely recognized. But programs could benefit from standardization and systematic recording by creating a European outcome register. This survey provides the basis for the development of a much-needed expert panel recommendation using for example a Delphi method, which may function as a controlled debate between a group of experts moving toward consensus.^43^ A clear evidence-based practice recommendation could foster collaboration across European pediatric heart centers and is urgently needed to support centers in the implementation of state-of-the-art neuromonitoring, neuroimaging, and follow-up to optimize care and thereby improve neurodevelopmental outcomes in infants with complex CHD.

## Supplementary information

Supplementary information

## Data Availability

The datasets analyzed during the current study are available from the corresponding author on reasonable request.
