# Supplementary material for: Neuromonitoring, neuroimaging, and neurodevelopmental follow-up practices in neonatal congenital heart disease: a European survey
Source: Pediatr Res. 2022 Apr 12;93(1):168–75. doi: 10.1038/s41390-022-02063-2 (PMC9876786; doi:10.1038/s41390-022-02063-2)
Supplement: Supplementary file 1 — Supplementary information [file 41390_2022_2063_MOESM1_ESM.pdf]

European **A**ssociation **B**rain and **C**ongenital Heart Disease

HeartSurvey2019 → Neuromonitoring_Survey 16.05.2020, 09:53

Seite 01

Intro

The **European Association Brain and Congenital Heart Disease (ABC) Consortium** aims to improve the neurodevelopmental outcome of infants with severe congenital heart disease. For this, we are interested in how neuromonitoring and neurodevelopmental care is practiced across European centres.

We would be very grateful if you could take the time to fill in the following 10 minutes questionnaire. If you need to obtain detailed information from your colleagues, the questionnaire can be paused and continued later.

We aim to analyse the information obtained from this questionnaire in order to issue guidelines regarding optimal neuromonitoring and neurodevelopmental care in infants with congenital heart disease.

We will analyse your responses anonymously, however if you wish you can provide your contact details at the end of the survey so we could potentially contact you for further information.

Thank you very much for your help!

Bea Latal, Manon Benders, Serena Counsell and Bettina Reich on behalf of the European ABC

This initiative is funded by the European Society for Paediatric Research

Seite 02

BG

1. Please give us some information on your institution.

This is important for us to identify multiple reports from the same site.

Hospital	
Unit	

2. What is your profession?

- Consultant Neonatologist
- Consultant in Paediatric Intensive Care Medicine
- Consultant Paediatrician
- Consultant Paediatric Cardiology
- Other Please specify

Seite 03

BG2

3. Where are neonates and infants in general treated prior to cardiopulmonary bypass surgery?

Neonatology department

Cardiology department

Paediatric intensive care unit

Other [Please specify]

4. Is the above mentioned unit rather a intensive care or high dependency/ intermediate care unit?

Intensive care

High dependency unit

Other [Please specify]

5. Are you involved in the care of patients with congenital heart disease undergoing neonatal cardiac surgery?

(excluding patent ductus arteriosus ligation)

Yes

No

Other [Please specify]

6. How many paediatric (0-16 years of age) surgeries are approximately being performed in your centre per year?

[Surgeries/year]

7. How many neonates (< 28 days) with complex congenital heart disease receive cardiopulmonary bypass (CPB) surgery in your institution per year?

CPB

[Please choose]

8. How many neonates (< 28 days) with complex congenital heart disease receive hybrid procedure (Giessen approach) in your institution per year?

Hybrid

[Please choose]

Seite 04

PreM

9. What type of neuromonitoring do you use in neonates prior to cardiopulmonary bypass surgery?

Amplitude integrated EEG [Please specify aEEG machine or write unknown]

Near infrared spectroscopy

Conventional EEG

None

Seite 05

PreM2

10. Do you use 1-channel or 2-channel aEEG?

1-channel

2-channel

11. How do you use conventional EEG?

Continuous

Non-continuous

Video EEG

12. Do you use a neonatal or adult/paediatric near infrared spectroscopy sensor?

Neonatal sensor

Paediatric/adult sensor

Don’t know

13. How is monitoring performed?

Routinely

Clinical indication

Research

Seite 06

PreIm

14. Which of these imaging methods do you use?

Cranial ultrasound

Cerebral MRI

Cerebral CT

None

Seite 07

PreIm2

15. Cerebral MRI before surgery is used in

Symptomatic patients

Clinical routine [If clinical routine, for which patients?]

Research/study protocol

16. Do you routinely perform cerebral MRI also for infants on the intensive care unit?

Yes

No

17. Would you like to comment on your answer above?

e.g. feasibility, concerns, strategies

[ ]

18. How do you usually perform cerebral MRI before surgery?

Natural sleep (feed and sleep)

Sedation [Please specify drug]

General anaesthesia [Please specify drug]

Seite 08

PreN

19. Do you perform a standardized neurological examination (e.g. Hammersmith, General Movement Assessment) on neonates prior to surgery?

Routinely

As part of research protocol

Only if clinical concern

No

Seite 09

PreN2

20. Who performs the neurological examination?

Child neurologist

Neonatologist

Developmental paediatrician

Physical therapist

Other Please specify

Seite 10

CPB

21. What type of neuromonitoring do you use during cardiopulmonary bypass surgery?

Near infrared spectroscopy

Amplitude integrated EEG

Bispectral index

EEG

None

22. Do you use neuroprotective therapies during cardiopulmonary bypass surgery?

(e.g. Allopurinol, Erythropoetin etc.)

No

Yes Please specify

Don’t know

Seite 11

CPB2

23. In which case do you administer the neuroprotective drug?

As part of study protocol

Clinical routine

Other Please specify

Seite 12

PostM

24. Do you measure biochemical parameters after cardiopulmonary bypass surgery?

Neuron specific enolase (NSE)

Protein S100

Glial fibrillary acidic protein (GFAP)

Other [Please specify]

None

Don’t know

25. What type of neuromonitoring do you routinely use in neonates during the first 48 hours after cardiopulmonary bypass surgery?

Amplitude integrated EEG [Please specify aEEG machine or write unknown]

Near infrared spectroscopy

Continuous EEG

None

Seite 13

PostIm

26. Which of these imaging methods do you use after cardiopulmonary bypass surgery?

Cranial ultrasound

Cerebral MRI

Cerebral CT

None

Seite 14

PostIm2

27. Cerebral MRI after surgery is used in

Symptomatic patients

Clinical routine [Please specify for which patients]

Research/study protocol

Other

[Please specify]

Seite 15

PostIm3

28. At which day after cardiopulmonary bypass surgery do you usually perform cerebral MRI?

Approximately	[ ] days after surgery	Don’t know

29. How do you perform cerebral MRI after cardiopulmonary bypass surgery?

Natural sleep (feed and sleep)

Sedation [Please specify drug]

General anaesthesia [Please specify drug]

30. Are MR venography or susceptibility-weighted imaging (SWI) routinely performed?

Venography

SWI

None

Don’t know

Seite 16

PostN

31. Do you perform a standardized neurological examination (e.g. Hammersmith, General Movement Assessment) on neonates prior to discharge?

Routinely

As part of research protocol

Only if clinical concern

No

Seite 17

PostN2

32. Who performs the neurological examination?

Child neurologist

Developmental paediatrician

Physical therapist

Other [Please specify]

Seite 18

PostFU

33. Does your centre have a formal process in place to identify and follow-up patients after cardiopulmonary bypass surgery for neurodevelopmental assessment?

Yes [Please specify at what ages patients are followed up]

In planning

No

Seite 19

PostFu2

34. Do you have a register for the patients in neurodevelopmental follow up?

Yes

No

35. Do you believe that an on-going register of neonates with congenital heart disease undergoing cardiopulmonary bypass surgery should be implemented?

Yes

No

36. Would you be interested in joining a European neurodevelopmental outcome register for neonates with congenital heart disease undergoing cardiopulmonary bypass surgery?

Yes

No

Don’t know

37. Would you like to comment on the current implementation of a register or on planning to start one?

38. We would appreciate if you would provide your contact information for further inquiries

this is optional

Your name

Email address

Letzte Seite

Thank you for completing this questionnaire!

We appreciate you took your time and contributed to our research. Your answers were transmitted, you may close the browser window or tab now.

For further comments, inquiries or feedback please feel free to contact us: HeartSurvey@kispi.uzh.ch

Bea Latal, Manon Benders, Serena Counsell and Bettina Reich on behalf of the European ABC

Einladung zum SoSci Panel

Das nicht-kommerzielle SoSci Panel würde Sie künftig gerne zu interessanten Onlinebefragungen einladen. Wir würden uns sehr freuen, wenn Sie die wissenschaftliche Forschung durch Ihre Teilnahme unterstützen.

E-Mail: ____________________ Am Panel teilnehmen

Die Teilnahme am SoSci Panel ist freiwillig und kann jederzeit widerrufen werden, Sie gehen mit der Teilnahme keinerlei Verpflichtungen ein.

Wenn Sie Ihre E-Mail-Adresse eintragen, erhalten Sie zunächst eine Bestätigungs-Mail. In dieser E-Mail finden Sie einen Link, um die Teilnahme am SoSci Panel zu bestätigen sowie weitere Informationen zum strengen Datenschutz im SoSci Panel.

Wir senden Ihnen selbstverständlich keine Werbung und geben Ihre E-Mail-Adresse nicht an Dritte weiter.

Der Fragebogen, den Sie gerade ausgefüllt haben, wurde gespeichert. Sie können das Browserfenster selbstverständlich auch schließen, ohne am SoSci Panel teilzunehmen.

contact: HeartSurvey@kispi.uzh.ch – 2019
